# Maternal metabolic health conditions and risk of stillbirth in India: evidence from a nationwide survey

**DOI:** 10.1186/s12889-025-23617-z

**Published:** 2025-07-09

**Authors:** Mohammad Hammad, Shivalingappa Halli, Mohammad Hifz Ur Rahman

**Affiliations:** 1 Department of Community Medicine, Manipal Tata Medical College, Manipal Academy of Higher Education, Manipal, India; 2 College of Community and Global Health, Rady Faculty of Health Sciences, University of Manitoba, Winnipeg, MB Canada; 3 Present Address: Department of Public Health, College of Medicine and Health Sciences, National University of Science and Technology, Sohar, Oman

**Keywords:** Stillbirth, Hypertension, Diabetes, National family health survey, India, Metabolic health

## Abstract

**Background:**

Stillbirth, defined by foetal death at or beyond 28 weeks of gestation, represents a significant challenge in India, contributing to approximately 500,000 foetal deaths each year. The country’s stillbirth rate of 12.2 per 1000 births underscores the imperative to address this preventable occurrence. While maternal metabolic conditions diabetes, and hypertension, are widely recognized as established risk factors for stillbirth worldwide, the extent of their impact on India’s stillbirth burden remains inadequately elucidated due to limited evidence.

**Methods:**

This cross-sectional study utilized NFHS-5 data to examine stillbirths in the most recent pregnancy outcomes of 204,723 women aged 15–49 years, sampled from all states and union territories of India. The primary exposures assessed were diabetes, and hypertension. Descriptive analyses were conducted to determine the prevalence of diabetes, hypertension and stillbirths. Logistic regression was used to quantify the association between diabetes, hypertension and the risk of stillbirth, indicated by adjusted odds ratios (AOR) with 95% confidence intervals (CI). The study also assessed effect modification by maternal age, education, wealth quintile, and social category.

**Results:**

The prevalence of diabetes and hypertension was 1% and 3% respectively, while the stillbirth rate was 1%. diabetes conferred a significantly higher risk of stillbirth with an increase of 74% (AOR 1.74, CI 1.14–2.67) as compared to women without diabetes. The risk was potential among mothers with hypertension with an increase of 50% (AOR 1.50, CI 1.16–1.95) on contrary to women without hypertension. The combined model (i.e. having diabetes or hypertension) also showed a significant risk of stillbirth with a higher risk of 58% (AOR 1.58, CI 1.25–1.99) indicating a synergistic interaction. Stratified analyses revealed the stillbirth risk among mothers belonging to the scheduled caste category (AOR 1.30, CI 1.10–1.53).

**Conclusion:**

Diabetes, and hypertension, increase stillbirth risk in India, highlighting the need for better metabolic health management pre- and during pregnancy. Our research highlights the need of integrated care for diabetes and hypertension is crucial. Targeted interventions for high-risk mothers and improved screening are vital to reduce stillbirth rates. More research is needed to understand these risks better. Collaboration across medical fields is essential to save lives and improve pregnancy outcomes.

**Supplementary Information:**

The online version contains supplementary material available at 10.1186/s12889-025-23617-z.

## Introduction

At 1.9 million stillbirths globally per year- one every 16 s, the burden of stillbirths continues to be a significant public health concern [1]. More than 40% of the stillbirths occur during labour which can be prevented with better quality of healthcare during childbirth [2]. The global stillbirth rate in 2021 was 13.9 per 1000 live births. Sub-Saharan Africa and South Asia accounted for three-quarters of all stillbirths, with a notable concentration of stillbirths in these regions with detrimental effects on communities as well as the emotional toll on impacted families. Healthcare providers and the economy highlight the need to address the issue urgently [3].

Even though stillbirths have a significant impact, they have not received the same amount of concern from the community as maternal and child mortality on the global health agenda [3–5]. However, there were no targets in the 2030 Agenda for Sustainable Development Goals explicitly concerning stillbirth.

In India, the prevalence of hypertension and diabetes among pregnant women is a growing concern. The National Family Health Survey-4 data revealed that 4.4% women had hypertension, and 2.4% women diagnosed with diabetes [6]. The increase in gestational diabetes mellitus (GDM) from 0.53% in 2015-16 to 0.80% in 2019-21 at the national level is alarming and can lead to adverse pregnancy outcomes such as preterm birth, stillbirth, and maternal mortality [7]. These conditions not only pose risks to the health of the mother but also have potential long-term effects on the child.

In India, the impact of the issue is particularly alarming, with an estimated 286,482 stillbirths in 2021, consistently placing the country first in the absolute number of stillbirths for over two decades [1, 2, 5]. This is despite the remarkable progress made by the county in reducing stillbirth rates over the past twenty years, with rates decreasing from 29.6 per 1000 births in 2000 to 12.2 per 1000 births in 2021 [5]. Recognizing stillbirths as a persistent public health challenge, India launched the India Newborn Action Plan (INAP) in 2014 with an ambition to reduce the stillbirth rate to less than 10 per 1000 births by the year 2030 [8].

Previous research has mainly focused on the factors like pre-term birth [9, 10], post neonatal mortality [11], infant mortality [12] and other maternal factors but studies on association of stillbirth with maternal metabolic conditions during pregnancy have not been done at a nationally representative level. In the present study we will focus on the two metabolic conditions of women during pregnancy diabetes and hypertension respectively and will understand the risk poised to the foetus and the mothers suffering from these metabolic diseases with confounder bias by including risk factors like age of women, source of water, type of delivery, facility at birth as well as adjusting of socio-demographic factors. Although there are various risk factors in stillbirth the study exclusively focuses om diabetes and hypertension due to their increasing prevalence in India and there established biological plausibility in affecting foetal outcomes.

## Methods

### Data

Our analysis is based on individual-level data sourced from the National Family Health Survey 5 (NFHS-5) conducted in India from 2019 to 2021. The National Family Health Surveys (NFHS) constitute a series of cross-sectional, nationally representative surveys designed to capture information on various demographic, socioeconomic, maternal, and child health indicators, as well as reproductive health and family planning aspects. Employing a two-stage stratified sampling approach, the fifth round of NFHS involved interviewing approximately 724,115 women aged 15 to 49 from 636,699 households, achieving a commendable 92% response rate. Additional details regarding the sampling strategy and tools employed can be utilized elsewhere. Individual Recode file (IAIR7EFL) was utilized for the analysis. The study sample was 204,723 which consisted of women aged 15–49 years who had their pregnancy in the past 5 years preceding the survey [13].

### Preliminary definitions

#### Outcome variable

The primary outcome variable for the study is stillbirth of the child i.e. a foetus which dies after 28 weeks of pregnancy, before or at the time of birth, is considered as a stillbirth. Stillbirth was calculated using the calendar data. Calendar data provides more robust estimation of the stillbirth [14]. Miscarriages and abortions were excluded from the analysis.

### Independent variables

The primary exposure variable under examination were the maternal metabolic conditions having diabetes categorized in two groups: diabetic and non-diabetic, having hypertension classified into two categories: hypertensive and non-hypertensive. Socio-demographic attributes encompassed the age, educational level, wealth index and place of residence and social category of the respondent. The household characteristics were also included in the study and were composed of type of sanitation facility (unimproved or improved), type of water source (piped improved, other improved and unimproved source). Maternal factors such as facility at birth (public, private or other), type of delivery (caesarean or non-caesarean), number of antenatal visits (no visits, 1 to 4 Visits, more than 4 visits) were also considered for the study.

The wealth index is a composite indicator of a household’s standard of living, derived using principal component analysis (PCA) based on data on asset ownership, housing quality, and access to basic services. In NFHS, households are typically classified into five quintiles; however, for this study, they were re-categorized into three groups: Poor (poorest and poorer), Middle, and Rich (richer and richest). This index serves as a widely accepted proxy for economic status and forms a key component of socioeconomic status (SES) in population-based surveys like NFHS.

### Statistical analysis

Univariate analysis was utilized to understand the sample characteristics of the women who had their births in last 5 years along with background variables. The bivariate analysis along with chi-square test of association was employed to understand the distribution of stillbirth with two metabolic conditions diabetes and hypertension. The strength of bivariate analysis along with chi-square test of association lies in its ability to determine whether is a statistically significant association between the variables. It allows to assess the independence of variables without assuming a normal distribution.

The relationship between stillbirth and maternal metabolic conditions was accessed using the binary logistic regression model.

The binary logistic regression model offers several strengths, including its ability to predict binary outcomes with strong interpretability through odds ratios. It efficiently handles non-linear relationships between the independent variables and the probability of the outcome. Additionally, the model is robust to non-normality in the predictors, making it suitable for a wide range of data types.

An aggregate variable was created for the metabolic conditions with women suffering from the two conditions namely diabetes, hypertension were clubbed into a single variable in order to run the binary logistic regression model and understand the combined effect of the diseases along with all the covariates i.e. maternal and socio-demographic factors.

A thorough assessment of multicollinearity was done using the Variance Inflation Factor (VIF). After performing the VIF Analysis, all variables included in the model displayed the VIF below 5, indicating that multicollinearity is not a concern in the model, as per commonly accepted guidelines. Therefore, the regression results are stable, and the interpretation of the coefficients are reliable. In addition to the VIF analysis, we also examine the correlation matrix of our independent variables. This further confirmed that multicollinearity was not a significant concern in the models. The tables for VIF and correlation matrix are present in Tables 1 and 2 of the supplementary material. The selection of the covariates was informed by existing literature, which identifies both pertinent risk factors and potential confounders that may influence the association between metabolic conditions and stillbirth [7, 11, 12, 15, 17].

**Table 1:** Sample background characteristics and prevalence of stillbirth with chi-square test of significance among women aged 15–49 years in India during 2019-21

Variables	Sample	Percent	Stillbirths (w%)^a^	*p*-value
Having diabetes				
Not having diabetes	200,840	99.01	1.55	0.01
Having diabetes	2009	0.99	3.24	
Having hypertension				
Not having hypertension	196,721	96.71	1.53	0.01
Having hypertension	6695	3.29	2.65	
Having diabetes or hypertension				
Not having diabetes or hypertension	196,632	96.05	1.52	0.01
Having either diabetes or hypertension	8091	3.95	2.77	
Type of place of residence				
Urban	45,025	21.99	1.29	0.01
Rural	159,698	78.01	1.66	
Wealth Category				
Poor	97,669	47.71	1.82	0.01
Middle	40,229	19.65	1.56	
Rich	66,825	32.64	1.24	
Highest level of Education				
No education	41,978	20.5	1.95	0.01
Primary	25,351	12.38	1.69	
Secondary	106,987	52.26	1.52	
Higher	30,407	14.85	1.13	
Social category				
Scheduled caste	40,601	19.83	1.37	0.01
Scheduled tribe	40,702	19.88	1.98	
Other backward classes	77,335	37.78	1.46	
General/Upper Caste	46,085	22.51	1.48	
Region				
Northern	28,907	10.55	1.06	0.01
Central	53,747	27.38	1.98	
Eastern	39,153	25.74	1.99	
Northeastern	31,730	4.18	1.16	
Western	17,895	12.87	1.09	
Southern	33,291	19.28	1.07	
Type of Water Source				
Unimproved Source	13,396	6.99	1.2	0.01
Improved/Piped Source	86,395	45.06	1.28	
Other Improved Source	91,960	47.96	1.77	
Age of Woman				
20 years and below	25,098	13.77	1.65	0.01
21–30 years	141,754	71.85	1.27	
30 years & above	33,014	14.38	0.96	
Facility at birth				
Public	114,951	61.86	0.95	0.01
Private	40,280	28.19	1.28	
Other	21,611	9.94	1.26	
Number of antenatal visits				
No Visits	41,703	20.12	4.04	0.01
1 to 4 visits	89,110	42.4	1.14	
More than 4 visits	73,910	37.48	1.01	
Whether had birth by caesarean method				
No	139,083	78.65	0.98	0.01
Yes	37,759	21.35	1.36	
N	204,723	100.00	2596 (1.56)	

^a^% weighted

**Table 2:** Unadjusted logistic regression model estimates for risk of stillbirth by primary exposure variables in India during 2019-21

Variables	Unadjusted Model
	OR^a^ (95% CI)
Having Diabetes	
Not having diabetes (ref.)	Ref.
Having diabetes	2.17*(1.65–2.88)
Having Hypertension	
Not having hypertension (ref.)	Ref.
Having hypertension	1.98*(1.68–2.35)
Having Diabetes or Hypertension	
Not having diabetes or hypertension (ref.)	Ref.
Having either diabetes or hypertension	2.05*(1.76–2.39)

^a^Odds Ratio, *- *p *< 0.05

We employed a stepwise modelling approach to systematically investigate the independent and combined effects of the metabolic conditions- namely diabetes and hypertension- on stillbirth. By isolating diabetes in the first model and hypertension in the second, we aimed to assess their individual contributions to stillbirth risk. The third model, which considered the presence of either condition, was designed to capture cumulative or interactive effect of the metabolic conditions. This approach allows for a clearer understanding of how each condition independently and jointly influences the adverse pregnancy outcomes.

## Results

Table 1 shows the sample characteristics of women aged 15–49 along with background variables. 3.29% percent of women had hypertension, and 1% women had diabetes. 78% of the women lived in rural areas, 20.5% of the women were illiterate and only 14.9% of women had attained higher education and nearly half of the women belonged to poor households (47.7%). Maternal characteristics showed most of the births happened to women in the age group 21–30 years (70.9%).

Table 1 also presents the prevalence of stillbirths across the primary exposure variable and other sociodemographic and environmental factors. Women with diabetes and hypertension have a higher prevalence of stillbirth (3.2% and 2.7%, respectively) compared to those who are not. Stillbirths are higher in rural women (1.7%) compared to those residing in urban areas (1.3%). The prevalence of stillbirth decreases with increased wealth and the level of education women attain. The prevalence of stillbirth is significantly lower among women who get 4 or more antenatal visits (1%) compared to those with no antenatal visits (around 4%).

**Fig. 1:**
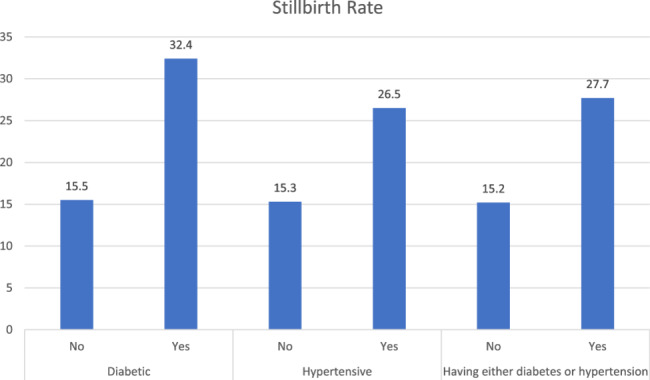
Stillbirth rate (per 1000) for metabolic conditions under examination i.e. diabetes and hypertension in India during 2019-21

Figure 1 shows the stillbirth rates (per 1000) for women suffering from diabetes, hypertension, and a combined rate for either of the diseases. Surprisingly, there is a significant difference of stillbirth rate among women who are suffering from these metabolic conditions during their pregnancy as compared to the women who are not. Diabetic women had a stillbirth rate of 31 stillbirths per 1000 whereas non-diabetic women have 14.5 per 1000 total births. Similarly, women having hypertension had stillbirth rate around 28 stillbirths per 1000 total births, which is significantly higher than those who do not have hypertension.

Table 2 presents the unadjusted odds ratios for the primary exposure variables for the study. Women who had diabetes had almost two times more likelihood of suffering a stillbirth (OR 2.17; 1.65–2.88, 95% CI) similarly a synergistic relation was seen with women who had hypertension with nearly double the risk of suffering a stillbirth (OR 1.98; 1.68–2.35, 95% CI). Having any one of the metabolic conditions i.e. diabetes or hypertension during also came with similar odds with almost double the risk of stillbirth (OR 2.05; 1.76–2.39, 95% CI).

Table 3 provides the logistic regression estimates for risk of stillbirth adjusted with background variables.

**Table 3:** Adjusted logistic regression model estimates for risk of stillbirth by background characteristics among women aged 15–49 years in India during 2019-21

Variables	Model-1^a^	Model-2^b^	Model-3^c^
	AOR^d^(95% CI)	AOR (95% CI)	AOR (95% CI)
Having diabetes			
Not having diabetes (ref.)	Ref.		
Having diabetes	1.74*(1.14,2.67)		
Having hypertension			
Not having hypertension (ref.)		Ref.	
Having hypertension		1.50*(1.16,1.95)	
Having diabetes or hypertension			
Not having diabetes or hypertension (ref.)			Ref.
Having either diabetes or hypertension			1.58*(1.25,1.99)
Type of place of residence			
Urban (ref.)	Ref.	Ref.	Ref.
Rural	1.19*(1.02,1.40)	1.18*(1.01,1.39)	1.18*(1.01,1.39)
Highest level of Education			
No education (ref.)	Ref.	Ref.	Ref.
Primary	1.01(0.85,1.20)	1.02(0.85,1.21)	1.01(0.85,1.20)
Secondary	0.85*(0.74,0.98)	0.85*(0.75,0.99)	0.85*(0.74,0.98)
Higher	0.72*(0.59,0.89)	0.73*(0.59,0.90)	0.72*(0.58,0.89)
Wealth Category			
Poor (ref.)	Ref.	Ref.	Ref.
Middle	0.79*(0.68,0.92)	0.79*(0.68,0.92)	0.80*(0.69,0.93)
Rich	0.64*(0.54,0.76)	0.64*(0.54,0.76)	0.65*(0.55,0.78)
Social category			
General/Upper Caste (ref.)	Ref.	Ref.	Ref.
Scheduled caste	1.29*(1.09,1.53)	1.30*(1.10,1.53)	1.30*(1.10,1.53)
Scheduled tribe	0.93(0.78,1.12)	0.94(0.79,1.14)	0.95(0.79,1.14)
Other Backward Classes	1.09(0.94,1.29)	1.10(0.95,1.28)	1.10(0.95,1.28)
Region			
Northern (ref.)	Ref.	Ref.	Ref.
Central	1.86*(1.52,2.27)	1.89*(1.54,2.31)	1.88*(1.54,2.30)
Eastern	1.91*(1.55,2.36)	1.93*(1.57,2.39)	1.92*(1.56,2.37)
Northeastern	0.90(0.70,1.18)	0.94(0.73,1.22)	0.93(0.72,1.20)
Western	1.05(0.80,1.37)	1.06(0.81,1.40)	1.06(0.81,1.38)
Southern	1.05(0.83,1.34)	1.08(0.85,1.37)	1.07(0.84,1.35)
Type of Water Source			
Unimproved Source (ref.)	Ref.	Ref.	Ref.
Improved/Piped Source	1.05(0.84,1.33)	1.03(0.82,1.31)	1.02(0.81,1.28)
Other Improved Source	1.07(0.86,1.35)	1.06(0.85,1.37)	1.05(0.84,1.31)
Age of Woman			
21–30 years (ref.)	Ref.	Ref.	Ref.
20 years and below	1.04(0.88,1.22)	1.03(0.88,1.21)	1.04(0.88,1.22)
30 years & above	0.83(0.67,1.02)	0.83(0.67,1.02)	0.82(0.67,1.01)
Facility at birth			
Public (ref.)	Ref.	Ref.	Ref.
Private	1.62*(1.41,1.87)	1.63*(1.41,1.87)	1.63*(1.42,1.87)
Other	1.02(0.87,1.21)	1.03(0.87,1.21)	1.04(0.88,1.23)
Number of antenatal visits			
No Visits (ref.)	Ref.	Ref.	Ref.
1 to 4 visits	1.21(0.98,1.50)	1.22(0.99,1.52)	1.23(0.99,1.52)
More than 4 visits	1.24(0.99,1.55)	1.25*(1.00,1.57)	1.26*(1.00,1.58)
Whether had birth by caesarean method			
No (ref.)	Ref.	Ref.	Ref.
Yes	1.40*(1.22,1.61)	1.39*(1.21,1.60)	1.39*(1.21,1.60)

^a^Model - 1 considers women who only have diabetes

^b^Model - 2 considers women who only have hypertension

^c^Model - 3 considers women who have either diabetes or hypertension

^d^Adjusted Odds Ratio, *- *p *< 0.05

Model 1 shows the logistic regression model of risk of stillbirth in women who only have diabetes along with background variables. The model results indicate the women who have diabetes had 74% higher likelihood of having a stillbirth (AOR 1.74; 1.14–2.67, 95% CI).

There was a significant urban-rural disparity with women residing in rural places having 19% higher risk (AOR 1.19; 1.02–1.40, 95% CI). Additionally, as the education level increased the risk of stillbirth dropped significantly with women having higher education with the lowest risk (AOR 0.72; 0.59–0.76, 95% CI) followed by women with secondary education (AOR 0.85; 0.74–0.98, 95% CI).

The EAG (Empowered Action Group) states mainly in the central (including Uttarakhand, Uttar Pradesh, Madhya Pradesh, and Chhattisgarh) and eastern (comprising Bihar, Jharkhand, West Bengal, Odisha, and Sikkim) regions of India had a nearly double likelihood of risk of stillbirth with odds rations (AOR 1.86; 1.52–2.27, 95% CI) and (AOR 1.91; 1.55–2.36, 95% CI) respectively.

Women delivering at private health facilities had higher risk of stillbirth (AOR 1.62; 1.41–1.87, 95% CI) as compared to women delivering at public health facilities. Additionally, women delivering through caesarean deliveries had 40% higher risk of stillbirth (AOR 1.40; 1.22–1.61, 95% CI) as compared to women who had normal/vaginal deliveries.

Model 2 presents the logistic regression model analysing the risk of stillbirth in women who only have hypertension along with confounding variables. The results of the model indicated that women who had only hypertension had 50% higher odds of having a stillbirth (AOR 1.50; 1.16–1.95, 95% CI). Results were similar to Model-1 for the background variables.

Model 3 shows the combined model with conditions, diabetes, or hypertension. The logistic regression model results indicated a 58% (AOR 1.58; 1.25–1.99, 95% CI) higher risk of stillbirth in women with metabolic conditions such as diabetes or hypertension compared to those without these conditions.

In terms of socio-demographic factors, women residing in rural areas faced a 18% (AOR (1.18; 1.01–1.39, 95% CI) more significant risk of stillbirth than their urban counterparts. Additionally, women with secondary and higher education levels had significantly lower chances (AOR 0.85; 0.74–0.98, 95% CI), (AOR 0.72;0.58–0.59, 95% CI) of experiencing stillbirth compared to those who were illiterate or had no formal education. Household wealth was significantly associated with the risk of stillbirth. Women from middle-income households had a lower likelihood of experiencing stillbirth compared to those from poor households (AOR: 0.80; 95% CI: 0.69–0.93), and the risk was even lower among women from richer households (AOR: 0.65; 95% CI: 0.55–0.78).

Geographically, the Central region of India (including Uttarakhand, Uttar Pradesh, Madhya Pradesh, and Chhattisgarh) had nearly 2 times higher likelihood of stillbirth (AOR 1.88;1.54–2.30, 95% CI). Similarly, the Eastern region (comprising Bihar, Jharkhand, West Bengal, Odisha, and Sikkim) had 1.92 times higher probability of stillbirth (AOR 1.92;1.56–2.37, 95% CI) compared to the northern region (Jammu & Kashmir, Ladakh, Punjab, Himachal Pradesh, and Rajasthan).

The facility at birth was also significant, with the child born in private facilities having a 63% higher risk of stillbirth (AOR 1.63; 1.42–1.87, 95% CI) than those born in public facilities. The type of delivery was another important factor, with women undergoing caesarean sections having a 39% higher chance of stillbirth (AOR 1.39; 1.21–1.60, 95% CI) compared to those who had vaginal deliveries.

## Discussion

Our Study showed that for the women to be inflicted with any of the metabolic condition during their pregnancy i.e. diabetes and hypertension, certainly increases significantly the risk of them suffering a stillbirth compared to who are not suffering from any of these metabolic diseases during their pregnancy. Stillbirth can be a very tragic event in the life of a mother as well it takes an emotional toll on the family of the women [15–17].

The results showed a skewed relationship of stillbirth with women belonging to the more deprived groups with risk of stillbirth being higher in the women who had no formal education, those residing in rural areas or the women who belonged to the scheduled caste group as compared to the more prosperous and advantaged women [18–20].

The region wise burden of stillbirth is consistent with previous research with the higher risk among women residing in the central and the eastern regions of the country [21–24]. These are useful findings for policy makers to develop targeted interventions in the high burden states.

Results also indicated that because of pregnancy complications, women giving birth by the caesarean delivery also face 39% higher risk of stillbirth and similar evidence can also be found in the published research [25].

Based on our findings, we recommend a targeted approach to address the risk of stillbirth associated with maternal diabetes and hypertension in India. Priority should be given to enhancing screening and management of these conditions, particularly in rural areas and among disadvantaged groups where stillbirth risk is higher. Integrated care pathways for pregnant women with diabetes and/or hypertension should be developed, ensuring close monitoring throughout pregnancy. We advocate for strengthening the capacity of healthcare providers, to effectively manage these metabolic conditions. Targeted health education campaigns are crucial to raise awareness about the risks associated with diabetes and hypertension during pregnancy, focusing on populations with lower educational attainment. Efforts should be made to improve access to quality antenatal care and increase the number of antenatal visits, particularly for high-risk women. Addressing the urban-rural disparity in stillbirth risk by improving healthcare infrastructure in rural areas is essential.

There are several limitations to consider when using NFHS data to calculate stillbirth rates. Firstly, NFHS surveys are cross-sectional in nature, meaning that they provide a snapshot of the population at a single point in time and may not capture changes in stillbirth rates over time. Additionally, NFHS surveys rely on self-reported data, which can be subject to recall bias and underreporting, particularly for sensitive topics such as stillbirths. Furthermore, NFHS surveys may not capture stillbirths that occur outside of health facilities, as these events may be less likely to be reported or recorded. There might be residual confounding in our study, given the observational nature of the data. While we have adjusted for key sociodemographic and clinical factors, there may be unmeasured confounders that could influence the associations we observed. Despite these limitations, NFHS data remain a valuable resource for understanding trends in stillbirth rates and informing public health policies and programs aimed at reducing stillbirths in India.

## Conclusion

Maternal diabetes and hypertension are significantly associated with heightened stillbirth risk in India, indicating the need to optimize metabolic health before and during pregnancy. A synergistic interaction between diabetes and hypertension underscores the importance of integrated management of coexisting conditions. Tailored interventions for mothers at high-risk including those with inadequate antenatal care and in higher burden regions can help mitigate the risk. Our findings highlight the urgent need to improve screening and management of maternal metabolic conditions as a pivotal public health strategy to curb India’s large stillbirth burden. This has implications for antenatal care policies and programs targeting modifiable metabolic risks. Further research can elucidate biologic pathways underlying these associations. Collaborative efforts across obstetrics, medicine and public health incorporating contextual factors are essential to transform pregnancy outcomes.

## Supplementary Information

Supplementary Material 1.

## Data Availability

The data that supports the findings of this study are available on request. The dataset used in the study is available in the public domain and can be accessed on a request from DHS at https://dhsprogram.com/Data/. Dataset and materials used in this study are available on request from the corresponding author mohammad.rahman@manipal.edu.
