# Supplementary material for: Maternal metabolic health conditions and risk of stillbirth in India: evidence from a nationwide survey
Source: BMC Public Health. 2025 Jul 9;25:2422. doi: 10.1186/s12889-025-23617-z (PMC12239305; doi:10.1186/s12889-025-23617-z)
Supplement: Supplementary file 1 — Supplementary Material 1. [file 12889_2025_23617_MOESM1_ESM.docx]

**Table 1: Variation Inflation Factors for primary exposure and confounder variables for each Model 1, Model 2 and Model 3**

| Model 1 | |  | Model 2 | |  | Model 3 | |
| --- | --- | --- | --- | --- | --- | --- | --- |
| Variable | VIF |  | Variable | VIF |  | Variable | VIF |
| **Having diabetes** |  |  | **Having hypertension** |  |  | **Having diabetes or hypertension** |  |
| Not having diabetes (ref.) |  |  | Not having hypertension (ref.) |  |  | Not having diabetes or hypertension (ref.) |  |
| Having diabetes | 1 |  | Having hypertension | 1.01 |  | Having either diabetes or hypertension | 1.01 |
| **Type of place of residence** |  |  | **Type of place of residence** |  |  | **Type of place of residence** |  |
| Urban (ref.) |  |  | Urban (ref.) |  |  | Urban (ref.) |  |
| Rural | 1.16 |  | Rural | 1.16 |  | Rural | 1.16 |
| **Highest level of Education** |  |  | **Highest level of Education** |  |  | **Highest level of Education** |  |
| No education (ref.) |  |  | No education (ref.) |  |  | No education (ref.) |  |
| Primary | 1.42 |  | Primary | 1.42 |  | Primary | 1.42 |
| Secondary | 1.89 |  | Secondary | 1.89 |  | Secondary | 1.89 |
| Higher | 1.73 |  | Higher | 1.73 |  | Higher | 1.73 |
| **Social category** |  |  | **Social category** |  |  | **Social category** |  |
| None of them (ref.) |  |  | None of them (ref.) |  |  | None of them (ref.) |  |
| Scheduled caste | 1.59 |  | Scheduled caste | 1.59 |  | Scheduled caste | 1.59 |
| Scheduled tribe | 1.77 |  | Scheduled tribe | 1.78 |  | Scheduled tribe | 1.78 |
| Other Backward Classes | 1.8 |  | Other Backward Classes | 1.80 |  | Other Backward Classes | 1.80 |
| **Region** |  |  | **Region** |  |  | **Region** |  |
| Northern (ref.) |  |  | Northern (ref.) |  |  | Northern (ref.) |  |
| Central | 2.32 |  | Central | 2.32 |  | Central | 2.32 |
| Eastern | 2.11 |  | Eastern | 2.11 |  | Eastern | 2.12 |
| Northeastern | 2.12 |  | Northeastern | 2.13 |  | Northeastern | 2.15 |
| Western | 1.55 |  | Western | 1.55 |  | Western | 1.55 |
| Southern | 1.83 |  | Southern | 1.83 |  | Southern | 1.83 |
| **Type of Water Source** |  |  | **Type of Water Source** |  |  | **Type of Water Source** |  |
| Unimproved Source (ref.) |  |  | Unimproved Source (ref.) |  |  | Unimproved Source (ref.) |  |
| Improved/Piped Source | 4.35 |  | Improved/Piped Source | 4.34 |  | Improved/Piped Source | 4.32 |
| Other Improved Source | 4.27 |  | Other Improved Source | 4.26 |  | Other Improved Source | 4.24 |
| **Age of Woman** |  |  | **Age of Woman** |  |  | **Age of Woman** |  |
| 21-30 years (ref.) |  |  | 21-30 years (ref.) |  |  | 21-30 years (ref.) |  |
| 20 years and below | 1.06 |  | 20 years and below | 1.06 |  | 20 years and below | 1.06 |
| 30 years & above | 1.08 |  | 30 years & above | 1.08 |  | 30 years & above | 1.08 |
| **Facility at birth** |  |  | **Facility at birth** |  |  | **Facility at birth** |  |
| Public (ref.) |  |  | Public (ref.) |  |  | Public (ref.) |  |
| Private | 1.3 |  | Private | 1.30 |  | Private | 1.30 |
| Other | 1.17 |  | Other | 1.17 |  | Other | 1.17 |
| **Number of antenatal visits** |  |  | **Number of antenatal visits** |  |  | **Number of antenatal visits** |  |
| No Visits (ref.) |  |  | No Visits (ref.) |  |  | No Visits (ref.) |  |
| 1 to 4 visits | 3.74 |  | 1 to 4 visits | 3.73 |  | 1 to 4 visits | 3.70 |
| More than 4 visits | 3.92 |  | More than 4 visits | 3.90 |  | More than 4 visits | 3.88 |
| **Whether had birth by caesarean method** |  |  | **Whether had birth by caesarean method** |  |  | **Whether had birth by caesarean method** |  |
| No (ref.) |  |  | No (ref.) |  |  | No (ref.) |  |
| Yes | 1.23 |  | Yes | 1.23 |  | Yes | 1.24 |
| Mean VIF | 2.02 |  | Mean VIF | 2.02 |  | Mean VIF | 2.01 |

**Table 2: Pairwise correlation matrix for Model-1**

|  | Having Diabetes | Place of Residence | Highest Level of Education | Social Category | Regions | Type of Water Source | Age of Woman | Facility at Birth | Number of ANC Visits | Whether had c-section |
| --- | --- | --- | --- | --- | --- | --- | --- | --- | --- | --- |
| Having Diabetes | 1.00 |  |  |  |  |  |  |  |  |  |
| Place of Residence | -0.01 | 1.00 |  |  |  |  |  |  |  |  |
| Highest Level of Education | 0.01 | -0.21 | 1.00 |  |  |  |  |  |  |  |
| Social Category | 0.01 | -0.10 | 0.15 | 1.00 |  |  |  |  |  |  |
| Regions | 0.00 | -0.09 | 0.14 | -0.01 | 1.00 |  |  |  |  |  |
| Type of Source of Water | 0.00 | 0.24 | -0.13 | -0.03 | -0.09 | 1.00 |  |  |  |  |
| Age of Woman | 0.03 | -0.06 | -0.10 | 0.01 | -0.03 | -0.02 | 1.00 |  |  |  |
| Facility at Birth | 0.00 | -0.02 | -0.06 | 0.02 | 0.05 | 0.05 | 0.07 | 1.00 |  |  |
| Number of Antenatal Visits | -0.01 | -0.07 | 0.15 | 0.04 | 0.08 | -0.08 | -0.01 | -0.13 | 1.00 |  |
| Whether had C-section | 0.02 | -0.15 | 0.23 | 0.09 | 0.15 | -0.11 | 0.04 | 0.04 | 0.16 | 1.00 |

**Table 3: Pairwise correlation matrix for Model-2**

|  | Having Hypertension | Place of Residence | Highest Level of Education | Social Category | Regions | Type of Water Source | Age of Woman | Facility at Birth | Number of ANC Visits | Whether had c-section |
| --- | --- | --- | --- | --- | --- | --- | --- | --- | --- | --- |
| Having Hypertension | 1.00 |  |  |  |  |  |  |  |  |  |
| Place of Residence | -0.01 | 1.00 |  |  |  |  |  |  |  |  |
| Highest Level of Education | 0.01 | -0.21 | 1.00 |  |  |  |  |  |  |  |
| Social Category | 0.01 | -0.10 | 0.15 | 1.00 |  |  |  |  |  |  |
| Regions | -0.02 | -0.09 | 0.14 | -0.01 | 1.00 |  |  |  |  |  |
| Type of Source of Water | -0.01 | 0.24 | -0.13 | -0.03 | -0.09 | 1.00 |  |  |  |  |
| Age of Woman | 0.03 | -0.06 | -0.10 | 0.01 | -0.03 | -0.02 | 1.00 |  |  |  |
| Facility at Birth | 0.01 | -0.02 | -0.06 | 0.02 | 0.05 | 0.05 | 0.07 | 1.00 |  |  |
| Number of Antenatal Visits | -0.02 | -0.07 | 0.15 | 0.04 | 0.08 | -0.08 | -0.01 | -0.13 | 1.00 |  |
| Whether had C-section | 0.03 | -0.15 | 0.23 | 0.09 | 0.15 | -0.11 | 0.04 | 0.04 | 0.16 | 1.00 |

**Table 4: Pairwise correlation matrix for Model-3**

|  | Having Diabetes or Hypertension | Place of Residence | Highest Level of Education | Social Category | Regions | Type of Water Source | Age of Woman | Facility at Birth | Number of ANC Visits | Whether had c-section |
| --- | --- | --- | --- | --- | --- | --- | --- | --- | --- | --- |
| Having Diabetes or Hypertension | 1.00 |  |  |  |  |  |  |  |  |  |
| Place of Residence | -0.01 | 1.00 |  |  |  |  |  |  |  |  |
| Highest Level of Education | 0.01 | -0.21 | 1.00 |  |  |  |  |  |  |  |
| Social Category | 0.01 | -0.10 | 0.15 | 1.00 |  |  |  |  |  |  |
| Regions | -0.02 | -0.09 | 0.14 | -0.01 | 1.00 |  |  |  |  |  |
| Type of Source of Water | -0.01 | 0.24 | -0.13 | -0.03 | -0.09 | 1.00 |  |  |  |  |
| Age of Woman | 0.04 | -0.06 | -0.10 | 0.01 | -0.03 | -0.02 | 1.00 |  |  |  |
| Facility at Birth | 0.01 | -0.02 | -0.06 | 0.02 | 0.05 | 0.05 | 0.07 | 1.00 |  |  |
| Number of Antenatal Visits | -0.02 | -0.07 | 0.15 | 0.04 | 0.08 | -0.08 | -0.01 | -0.13 | 1.00 |  |
| Whether had C-section | 0.04 | -0.15 | 0.23 | 0.09 | 0.15 | -0.11 | 0.04 | 0.04 | 0.16 | 1.00 |
